# Physiological Mechanism of Exogenous 5-Aminolevulinic Acid Improved the Tolerance of Chinese Cabbage (*Brassica pekinensis* L.) to Cadmium Stress

**DOI:** 10.3389/fpls.2022.845396

**Published:** 2022-05-26

**Authors:** Lijing Yang, Yue Wu, Xiaomin Wang, Jian Lv, Zhongqi Tang, Linli Hu, Shilei Luo, Ruidong Wang, Basharat Ali, Jihua Yu

**Affiliations:** ^1^College of Horticulture, Gansu Agricultural University, Lanzhou, China; ^2^Key Laboratory of Cell Activities and Stress Adaptations, Ministry of Education, School of Life Sciences, Lanzhou University, Lanzhou, China; ^3^Department of Agronomy, University of Agriculture Faisalabad, Faisalabad, Pakistan; ^4^Gansu Provincial Key Laboratory of Arid Land Crop Science, Gansu Agricultural University, Lanzhou, China

**Keywords:** 5-minolevulinic acid, cadmium stress, Chinese cabbage, ascorbate-lutathione cycle, photosy nthesis

## Abstract

The 5-aminolevulinic acid (ALA), a new type of plant growth regulator, can relieve the toxicity of cadmium (Cd) to plants. However, its mechanism has not been thoroughly studied. In the study, the roles of ALA have been investigated in the tolerance of Chinese cabbage (*Brassica pekinensis* L.) seedlings to Cd stress. The results showed that Cd significantly reduced the biomass and the length of the primary root of seedlings but increased the malondialdehyde (MDA) and the hydrogen peroxide (H_2_O_2_) contents. These can be effectively mitigated through the application of ALA. The ALA can further induce the activities of antioxidant enzymes in the ascorbate-glutathione (AsA-GSH) cycle under Cd stress, which resulted in high levels of both GSH and AsA. Under ALA + Cd treatment, the seedlings showed a higher chlorophyll content and photosynthetic performance in comparison with Cd treatment alone. Microscopic analysis results confirmed that ALA can protect the cell structure of shoots and roots, i.e., stabilizing the morphological structure of chloroplasts in leaf mesophyll cells. The qRT-PCR results further reported that ALA downregulated the expressions of Cd absorption and transport-related genes in shoots (*HMA2* and *HMA4*) and roots (*IRT1, IRT2, Nramp1*, and *Nramp3*), which resulted in the low Cd content in the shoots and roots of cabbage seedlings. Taken together, the exogenous application of ALA alleviates Cd stress through maintaining redox homeostasis, protecting the photosynthetic system, and regulating the expression of Cd transport-related genes in Chinese cabbage seedlings.

## Introduction

Cadmium (Cd) is widely distributed in nature and is highly toxic to plants (Chen et al., 2018). The Cd-toxicity is a complex phenomenon that negatively impacts plant growth and development by inducing oxidative stresses and affecting element uptake (Xu et al., 2016). It also enhances the contents of reactive oxygen species (ROS) activity, which leads to lipid peroxidation (María et al., 2007), disrupting the structure and function of proteins, and affecting the expression of genes that are encoding metal transcription factors (Giovanni et al., 2010). In response to heavy metal stresses, plants have evolved adaptive mechanisms, i.e., upregulating the antioxidant defense system. For example, Qin et al. (2018) reported that wheat (*Triticum aestivum* L.) seedlings were tolerant against Cd stress by increasing the activities of enzymes in the ascorbate-glutathione (AsA-GSH) cycle. The AsA-GSH cycle in plants plays a crucial role in combating Cd stress (Qin et al., 2018) and other stresses, such as drought (Kang et al., 2013), low light (Hu et al., 2019), salt stress (Wu et al., 2019), and chilling stress (He et al., 2021). The enzymes [dehydroascorbate reductase (DHAR), monodehydroascorbate reductase (MDHAR), glutathione reductase (GR), ascorbic acid peroxidase (APX), and ascorbic acid oxidase (ASO)] and the antioxidants [ascorbic acid (AsA) and reduced glutathione (GSH) in AsA-GSH cycle can effectively scavenge ROS induced by the environmental stresses (Li et al., 2010)]. The high ratio of AsA/dehydroascorbic acid (DHA) and GSH/oxidized glutathione (GSSG) is essential for scavenging ROS, and the regeneration of AsA and GSH depends on the activities of DHAR, MDHAR, and GR (Niu et al., 2017).

Several families of metal transporters are found to play a key role in Cd absorption and transport in plants (Clemens, 2006). For example, the *iron-regulated transporter 1* (*IRT1*) and the *natural resistance-associated macrophage protein 1* (*Nramp1*) mediate Cd uptake in plant roots (Vert et al., 2002). The *Heavy metal ATPase 2* (*HMA2*) and *HMA4* can transfer Cd from the root to shoot through the xylem (Frederic et al., 2004; Takahashi et al., 2012). The over-expression of *OsHMA2* can reduce the Zn and Cd levels in rice grains (Takahashi et al., 2012). Studies have identified that the IRT and *Nramp* transporters participate in Cd accumulation and transport processes, which is important for plant tolerance to heavy metals (Alexander et al., 2011; Chen et al., 2019). The latest research found that the hydrogen-rich water reduced the Cd accumulation by downregulating *IRT1* gene expression (Wu et al., 2021).

The Cd stress can also be alleviated by 5-aminolevulinic acid (ALA) in plants (Wang F. J. et al., 2018; El-Amier et al., 2019). The ALA is a key precursor metabolic intermediate in plants, animals, and bacteria, which has been discovered as a new plant growth regulator (Wu et al., 2019). Recently, it was reported that ALA can improve plant growth, photosynthetic assimilation, and gas exchange capacity under Cd stress (Ali et al., 2013a,b). Recently, it was found that ALA was involved in the regulation of gene expression involved in Na^+^ transporter in cucumber roots under salt stress, which reduces Na^+^ upward transport (Wu et al., 2019). However, it remains unknown about the mechanism of ALA that is involved in the cabbage tolerance to Cd stress, and whether ALA is involved in the transcriptional regulation of Cd uptake and transporter genes.

Chinese cabbage (*Brassica pekinensis* L.) is one of the most widely grown vegetables in China. Its productivity and quality are considerably decreased under Cd stress. Therefore, reducing the accumulation of toxic metals in edible parts of vegetables by regulating the antioxidant enzyme system and metal ion absorption and transport genes is of great significance. Therefore, the aim of this study was to investigate the protective mechanism of ALA in the Cd tolerance of cabbage, and especially, the regulative mechanism involved in Cd transport and distribution.

## Materials and Methods

### Plant Materials and Chemical Treatment

Chinese cabbage (*Brassica pekinensis* L. cv.) seeds (Huangfei) were purchased from Qingfeng Seed Service (Lanzhou, China). Seeds were surface-disinfected using 1.5% sodium hypochlorite for 15 min and washed five times with distilled water. Then, seeds were germinated in the dark for 48 h at 25 ± 1°C. At the two-leaf stage, the morphologically uniform seedlings were selected and plugged into plate holes on a plastic container (17 cm × 11 cm × 6 cm) containing half-strength of Hoagland’s nutrient solution [25 mM KNO_3_, 25 mM Ca(NO_3_)_2_⋅4H_2_O, 7.5 mM MgSO_4_⋅7H_2_O, 5 mM NaH_2_PO_4_⋅2H_2_O, 0.04 μM EDTA⋅Na-Fe, 23 μM H_3_BO_3_, 4.75 μM MnSO_4_⋅H_2_O, 0.4 μM ZnSO_4_⋅5H_2_O, 0.15 μM CuSO_4_⋅5H_2_O, and 0.008 μM (NH_4_)_6_Mo_7_O_2_⋅4H_2_O]. The seedlings were grown in the climate box at 26 ± 1/18 ± 1°C (day/night) with 75% humidity, 41.38 W/m^2^ light intensity, and a 12 h/12 h light period.

Fourteen-day-old Chinese cabbage seedlings were treated for 7 days with 50 μM CdCl_2_. At the same time, ALA (25 mg/L) was sprayed onto the leaves, and it was applied every 3 days. After treatment, the leaves and roots were frozen immediately in liquid nitrogen and stored at −80°C. At least 48 seedlings were used for each treatment and the experiments were repeated thrice.

### Determination of the Biomass, Gas Exchange, and Chlorophyll Fluorescence Parameters

After treatment, 21-day-old seedlings were harvested and photographed. Primary root length, dry weight (DW), and fresh weight (FW) were measured from every treatment.

The content of chlorophyll was calculated according to the for-mar described by Lichtenthaler and Wellburn (1983). Samples (0.200 g each) of Chinese cabbage leaves were extracted using 80% buffered aqueous acetone for determining the chlorophyll content (Porra et al., 1989). The absorbance of the supernatant was determined at 646 and 663 nm. The *V* values indicate the dissolved volume of the determined solution; the FW values indicate the fresh weight of the sample.


Chla(mggFW-1)=(12.21×OD-6632.81×OD)646×V/FW

Chlb(mggFW-1)=(20.13×OD-6465.03×OD)663×V/FW

Plant gas exchange parameters, including the net photosynthetic rate (Pn), intercellular CO_2_ concentration (Ci), stomatal conductance (Gs), and transpiration rate (Tr), were measured using the portable photosynthesis system (CIRAS-2, PP System, United Kingdom) according to the method described by Pimentel et al. (1999). Before determination, the seedlings were acclimated to light for 10 min in the culture environment. Then, the fourth functional leaf was selected and placed into the leaf chamber for determination. The conditions for Pn measurement were set as follows: the photosynthetic photon flux density, 400 μmol m^–2^ s^–1^; ambient CO_2_ concentration, 380 μmol mol^–1^; relative humidity, 70%, and leaf temperature, 25°C.

Chlorophyll fluorescence parameters of seedlings were measured using the modulated chlorophyll fluorescence imaging system (Maxi Imaging-PAM, Walz, Germany) (Gong et al., 2014). Before determination, the seedlings were adapted in darkness for at least 30 min. The fourth functional leaf of seedlings was selected for the determination. By applying a saturation pulse under 2,700 μmol m^–2^ s^–1^, the fluorescence parameters of minimum fluorescence (Fo) and maximum fluorescence yield (Fm) were obtained from dark-adapted leaves. The actinic light was adjusted to 81 μmol m^–2^ s^–1^, the leaves were light-adapted for 5 min, and opened every 20 s, lasting for 0.8 s. By applying actinic light, the indexes like minimum fluorescence (Fo’), steady chlorophyll fluorescence (Fs), and maximum fluorescence yield (Fm’) could be calculated. The actual photosynthetic efficiency (Fv/Fm) was calculated as described by Genty et al. (1989). The coefficient of actinic light quenching (qP) was calculated according to the method of Klughammer and Schreiber (2008). The specific computational formulas were as follows:


$$\mathrm{Fv}/\mathrm{Fm}=\left({\mathrm{Fm}}^{\prime }-\mathrm{Fs}\right)/{\mathrm{Fm}}^{\prime }$$

$$\mathrm{qP}=\left({\mathrm{Fm}}^{\prime }-\mathrm{Fs}\right)/\left({\mathrm{Fm}}^{\prime }-{\mathrm{Fo}}^{\prime }\right)\times {\mathrm{Fo}}^{\prime }/\mathrm{Fs}$$

### Ultrastructural Analysis of Chinese Cabbage

The leaves and roots were fixed with a buffer containing 2.5% glutaraldehyde in 0.1 M phosphate buffer (PBS, pH 7.4) for 24 h at 4°C. The samples were washed thrice with 0.1 M PBS (pH 7.4) and fixed in 1% H_2_OsO_4_ for 5 h at 4°C. Afterward, the samples were washed thrice with 0.1 M PBS (pH 7.4), and dehydrated using graded ethanol solutions (50, 70, 80, 90, and 100%) for 15 min every time; following this, the samples were acetone-infiltrated and embedded in Epon 812 epoxy resin. Ultrathin sections were cut on a microtome (Leica EM UC6 ultra-microtome, Japan), and stained with uranyl acetate and lead citrate for 15 min. Ultrathin sections of cabbage leaf and root were examined and photographed with the transmission electron microscope (TEM, JEOL JEM-1230, Japan). The electron microscope sample was processed according to the method described by the electron microscope center of Lanzhou University.

### Determination of Hydrogen Peroxide and Malondialdehyde Contents

The content of H_2_O_2_ was determined according to the method given by Gong et al. (2008). H_2_O_2_ content was measured using the ELISA kit (Beijing Solarbio Science & Technology Co., Ltd., Beijing, China) according to the instructions. The H_2_O_2_ content was calculated based on the standard curve.

The malondialdehyde (MDA) was determined according to the method of Zhang et al. (2007). The concentration of MDA was determined using the colorimetric method of thiobarbituric acid (TBA) with Micro MDA Assay Kit (BC0025; Beijing Solarbio Science & Technology Co., Ltd., Beijing, China) according to the manufacturer’s instructions.

### Determination of Antioxidant Enzyme Activities

Samples were homogenized in 5 ml of 50 mM PBS buffer (pH 7.8) at 4°C. The homogenate was centrifuged at 10,000 × *g* for 20 min at 4°C, and the supernatant was used to determine the enzyme activities (DHAR, MDHAR, GR, APX, and ASO). All the extraction methods of the enzyme have been slightly modified (Rao and Terry, 1989). The soluble protein concentration was determined by the method of Bradford (1976).

For dehydroascorbate reductase (DHAR) (EC 1.8.5.1) activity analysis, the reaction mixture included 50 mM PBS (pH 7), 2.5 mM GSH, and 0.1 mM DHA. The reaction was started by adding 50 μl of enzyme extract. The activity was measured using the decrease in absorbance at 265 nm in 1 min (Nakano and Asada, 1981).

For monodehydroascorbate reductase (MDHAR) (EC 1.6.5.4) activity analysis, the reaction mixture included 50 mM PBS (pH 7), 2 mM nicotinamide adenine dinucleotide phosphate (NADPH), and 2U ascorbate oxidase (AAO). The reaction was started by adding 90 μl of enzyme extract. The activity was measured using the decrease in absorbance at 340 nm in 1 min (Maria et al., 2000).

For glutathione reductase GR (EC 1.6.4.2) activity analysis, the reaction mixture included 0.1 M PBS (pH 7), 1 mM ethylenediaminetetraacetic acid (EDTA), 1 mM GSSG, 0.2 mM NADPH, and 50 μl of enzyme extract. The reaction was started by adding GSSG, and the decrease in absorbance was recorded at 340 nm (Foyer and Halliwell, 1976).

For ascorbic acid oxidase (ASO) (EC 1.10.3.3) activity analysis, the reaction mixture included 50 mM PBS (pH 7), 1 mM AsA, 1 mM EDTA, 2% povidone, 0.25% triton X-100, and 0.15 ml of enzyme extract in a final volume of 3 ml. The reaction was started by adding H_2_O_2_. The activity was calculated from the recorded decrease in absorbance at 265 nm for 1 min (Esaka et al., 1990).

For APX (EC 1.11.1.11) activity analysis, the reaction mixture included 50 mM PBS (pH 7), 0.5 mM AsA, 0.1 mM EDTA, 0.1 mM H_2_O_2_, and 30 μl of enzyme extract in a final volume of 2 ml. The reaction was started by adding H_2_O_2_. The activity was calculated from the recorded decrease in absorbance at 290 nm for 1 min (Nakano and Asada, 1981).

### Analysis of the Ascorbate-Glutathione Cycle

The total glutathione (GSH and GSSG) was extracted using an ELISA kit (Beijing Solarbio Science & Technology Co., Ltd., Beijing, China) according to the manufacturer’s instructions. Either leaf or root (0.100 g) was homogenized and centrifuged at 7,100 × *g* (total glutathione) or 1,100 × *g* (GSSG) at 4°C for 10 min. The supernatant was collected to determine total glutathione and GSSG.

The contents of AsA and DHA were analyzed according to the method as described by Arakawa et al. (1981). The AsA and DHA contents were analyzed according to the following steps. The first extract reagent (5 mL) was added to the frozen samples (0.1 g), and it was homogenized in an ice bath. Then, the homogenate was centrifuged at 8,000 × *g* at 4°C for 20 min for AsA or 16,000 × *g* at 4°C for 20 min for DHA, respectively. The analysis was performed using the AsA ELISA kit and DHA ELISA kit, respectively.

### Determination of Metal Concentrations

To determine the element contents, roots and leaves were separately harvested. The roots were washed thrice in distilled water. Then plant tissues were dipped in 20 mM EDTA for 30 s, following which they were washed with distilled water. The samples were then dried at 80°C to constant weight and grounded to a fine powder.

The content of Cd was measured according to the method as described by Wang F. J. et al. (2018). For determination of Cd content, the leaf and root samples (0.500 g) were put into a crucible on a hot plate until the initial smoke was observed and was burned to ash at 550°C in a muffle furnace. The ash samples were allowed to cool, and each was supplied with 10 ml of 6 M hydrochloric acid (HCl). Drops of 6 M HCl were added to dissolve the residues. Distilled water was then added to the mixture in a volumetric flask to the 50 ml mark. The Cd content was determined at 228.8 nm using the atomic absorption spectrophotometry method (ZEEnit 700P, Analytik Jena AG, Germany).

The contents of other elements were measured according to the method as described by Miria et al. (2016). For determination of other element contents, plant samples (0.500 g) were digested with 5 mL H_2_SO_4_ overnight. The samples were placed on a hot plate until the solution was brown. Ten drops of 30% H_2_O_2_ were slowly dropped into the solution. The operations were repeated thrice until the solutions were clear. Distilled water was then added to the mixture in a volumetric flask to the 50 mL mark. The concentrations of elements were determined using the atomic absorption spectrophotometer with the OD set as follows: 422.6 nm (Ca), 285.2 nm (Mg), 248.3 nm (Fe), 213.8 nm (Zn), and 278.4 nm (Mn).

### Gene Expression Measurement

The total RNA was isolated from Chinese cabbage tissues using a total RNA kit (Tiangen Biotech Co., Ltd., Beijing, China). The DNA-free total RNA (5 μg) was used for the first-strand cDNA synthesis in a 20 μl reaction volume (Thermo Fisher Scientific, MD, Lithuania) according to the manufacturer’s instructions. Real-time quantitative PCR (qRT-PCR) reactions were performed using the Bio-systems 7500 Real-Time PCR System (Applied Biosystems^®^, Foster City, CA, United States) with the SYBR Green intercalating dye fluorescence detection. The amplification program was as follows: 3 min at 95°C, 40 cycles of 5 s at 95°C, 10 s at 55°C. Relative gene expression was evaluated using the 2^–Δ^ ^Δ^ *^Ct^* method. The PCR primers were designed using Primer Premier 5 software (PREMIER Biosoft, Palo Alto, CA, United States), and is listed in Table 1. *Actin* (AF111812) was used as an internal control.

**TABLE 1:** The primer lists.

Gene name	Gene ID	5′→3′	3′→5′
*IRT1*	AY087095.1	TGGCATTCTTTTTCGCGGTG	GCCGAGCATGCATTGAGAAG
*IRT2*	BT025714.1	CTCGTCGACCTTCTGGCTAC	ACTTGGCGACGACAGACATT
*Nramp1*	AF165125.1	CCCCGAAGACCGTGCTAAAT	TACCCACCACGTTTCGTAGC
*Nramp3*	GC008629595.1	TCTTGATTGTTTCGTCTTC	TCCCATTGTAGCGATAAG
*HMA2*	NM119157.3	GAGGATGCCACATGGTTGGA	CTTTGGTACGGCGGAAGAGT
*HMA4*	AY096796	TTCCCCACAAGAATCGCTCC	CACTCGAACCTTCCACGTCA
Actin	AF111812	CCAGGAATCGCTGACCGTAT	CTGTTGGAAAGTGCTGAGGGA

### Statistical Analysis

Data are presented as the means ± SE. The data were analyzed by one-way ANOVA, which was conducted with the SPSS 19.0 (SPSS Inc., Armonk, NY, United States). Duncan’s multi-range test was performed to compare significant difference between treatments at *P* < 0.05. All the figures are completed by using the SigmaPlot 12.5.

## Results

### Effects of 5-Aminolevulinic Acid on the Biomass of Cabbage Under Cadmium Stress

**FIGURE 1:**
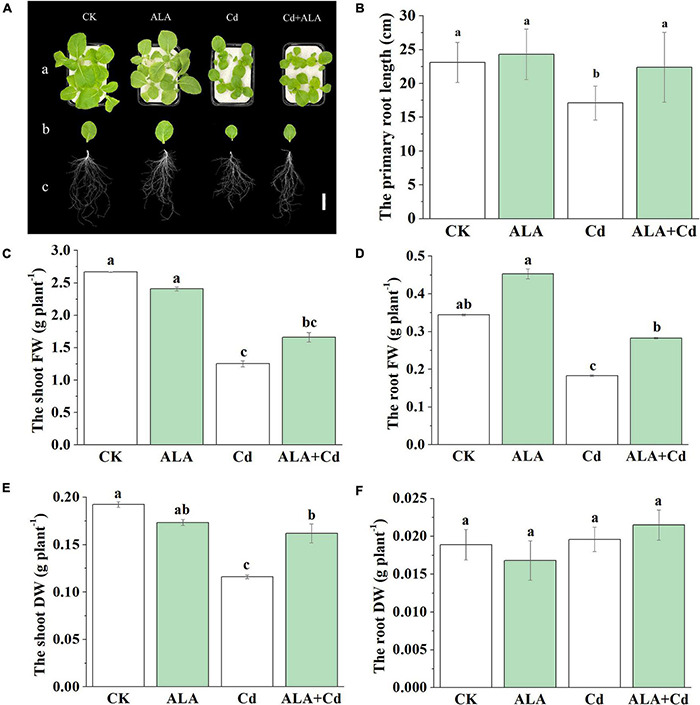
Exogenous ALA alleviated Cd stress-induced growth inhibition in Chinese cabbage. **(A)** Phenotypes of individual. Bar = 5 cm. **(B)** Changes in primary root length. Fresh weight (FW) of shoots **(C)** and roots **(D)**. Dry weight (DW) of shoots **(E)** and roots **(F)** The 14-day-old cabbage seedlings were transferred to 1/2 Hoagland medium containing 50 μM Cd for 7 days. About 25 mg/L 5-aminolevulinic acid (ALA) was sprayed onto the leaves, and it was applied every 3 days under ALA+Cd as well as ALA treatments. The mean ± SE are shown (*n* ≥ 3). Different letters indicate significant difference among the treatments (*P* < 0.05).

To examine the effects of ALA on Chinese cabbage seedlings exposed to Cd stress, we investigated the changes in seedling growth, root length, FW, and DW. Changes in the phenotype following Cd and ALA treatments are shown in Figure 1A. Compared to the control, the Cd stress significantly caused a decrease in the FW of roots and shoots and the length of the primary root (Figure 1). The FW of roots and shoots was similarly reduced by about 50% (Figures 1C,D). Based on the concentration curve of ALA, the biomass of Chinese cabbage seedlings at 25 mg/L of ALA reached the maximum under 50 μM Cd treatment (data not shown). Thus, the 50 μM Cd and 25 mg/L ALA were selected for treatment in the study. The DW of roots and shoots was also markedly decreased (Figures 1E,F). Under ALA + Cd treatment, the root length was increased by about 31% compared to that under Cd treatment alone (Figure 1B). The application of ALA increased the DW of shoots (about 40%) compared to that from Cd treatment alone, but not in the DW of roots (Figure 1F). Under normal conditions, the application of exogenous ALA had no significant effects on primary root length, FW, and DW of Chinese cabbage seedlings.

### Effects of 5-Aminolevulinic Acid on Chlorophyll Content and Photosynthetic Gas Exchange of Cabbage Seedlings

**FIGURE 2:**
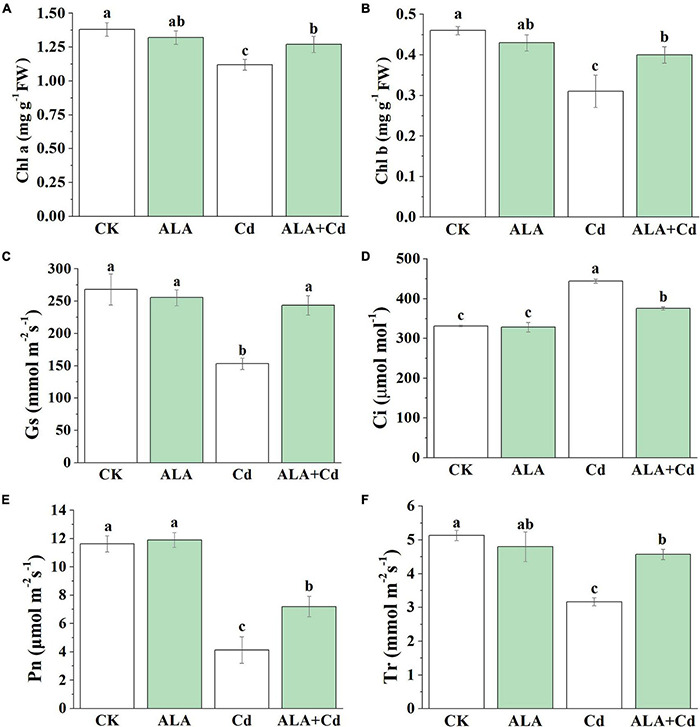
Effects of ALA under Cd stress on Chlorophyll content and photosynthetic gas exchange parameters of cabbage. **(A)** Chl *a*, **(B)** Chl *b*, **(D)** intercellular CO_2_ concentration (Ci), **(C)** stomatal conductance (Gs), **(E)** net photosynthetic rate (Pn), and **(F)** transpiration rate (Tr). The mean ± SE are shown (*n* ≥ 3). The seedlings were treated as in Figure 1. Different letters indicate significant difference among the treatments (*P* < 0.05).

Effects of ALA and Cd on chlorophyll content and photosynthetic gas exchange attributes are delineated in Figure 2. Results showed that the content of chlorophyll *a* (Chl *a*) and chlorophyll *b* (Chl b) under Cd stress were reduced by 19 and 33%, respectively. Under ALA + Cd treatment, compared with the single Cd treatment, the content of Chl *a* and Chl *b* was increased by 13 and 29%, respectively. Under ALA treatment alone, there was no significant difference between Chl *a* and Chl *b* content compared with the control.

Under Cd treatment, the gas exchange parameters were significantly decreased. The Pn, Gs, and Tr were decreased by 65, 42, and 38%, respectively, while Ci was significantly increased by 34%. Upon adding ALA under Cd stress, Pn, Gs, and Tr increased significantly by 74, 59, and 44%, respectively, and the Ci value decreased significantly by 16%. There is no significant difference for the above parameters between the ALA treatment alone and control conditions. The results showed that exogenous ALA could improve Pn under Cd stress, which was independent of stomatal structure.

### Effects of 5-Aminolevulinic Acid on Chlorophyll Fluorescence Parameters of Cabbage Under Cadmium Stress

**FIGURE 3:**
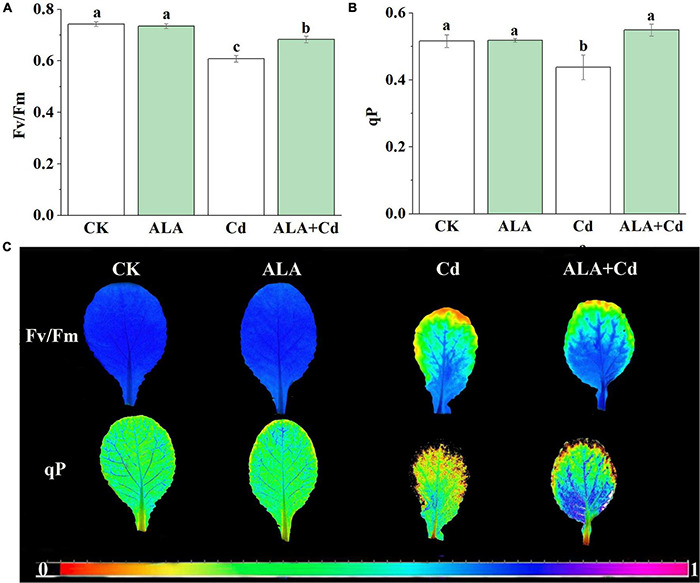
Effects of ALA under Cd stress on chlorophyll fluorescence parameters of cabbage. **(A)** The maximum PSII quantum yield (Fv/Fm), **(B)** photochemical quenching coefficient (qP) and **(C)** Chlorophyll fluorescence imaging. The seedlings were treated as in Figure 1. The mean ± SE are shown (*n* ≥ 3). Different letters indicate significant difference among the treatments (*P* < 0.05).

Chlorophyll fluorescence is regarded as an internal probe to provide insights into the relationship between plant photosynthesis and the environment. The Fv/Fm reflects the health status of the PSII system. The qP is the efficiency of light energy conversion and an important index for plants to absorb light energy, which reflects the rate of electron transfer in photosynthesis (Ivanov and Edwards, 2000). To further explore the mechanism by which ALA is involved in photosynthesis, the conversion efficiency of primary light energy of PS (Fv/Fm) and photochemical quenching coefficient (qP) were analyzed. Results stated that Fv/Fm and qP of cabbage leaves were decreased by 13 and 15%, respectively, under Cd stress (Figure 3A). They were significantly increased by 12 and 25%, respectively, under ALA + Cd co-treatment.

The fluorescence images of the Fv/Fm and qP are given in Figure 2C. The colors represent the absolute values of the ratio ranging from 0 (black) to 1 (purple). In the fluorescent image of qP, there were obvious orange spots in the serious stress area under Cd treatment, and the orange spots under co-treatment of ALA and Cd were less than those under Cd treatment alone. The color distribution of Fv/Fm under ALA and Cd co-treatment was more similar to the control treatment. The ALA alleviated the decrease of Fv/Fm under Cd stress, which indicated that ALA enhanced the light utilization of cabbage seedlings under Cd stress. The results showed that exogenous ALA could increase the electron transfer rate of PSII under Cd stress.

### Effect of 5-Aminolevulinic Acid on Ultrastructural and Morphometric Images of Cabbage Leaves and Roots

**FIGURE 4:**
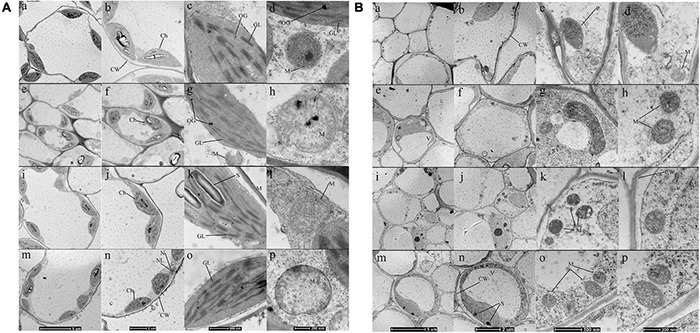
Effects of ALA under Cd stress on the ultrastructural observation of mesophyll cell and chloroplast of cabbage shoots **(A)** and roots **(B)**. In **(A,B)**: **(a,e,i,m)** (×1,900), **(b,f,j,n)** (×2,900), **(c,g,k,o)** (×13,000), **(d,h,l,p)** (×23,000); **(a–d)** Seedlings grown in normal condition. **(e–h)** Seedlings sprayed 25 mg/L ALA only. **(i–l)** 50 μM CdCl_2_ treated seedlings. **(m–p)** Seedlings simultaneously treated with 50 μM CdCl_2_ and 25 mg/L ALA. The seedlings were treated as in Figure 1. CW, cell wall; Ch, chloroplast; OG, osmiophilic globules; GL, grana lamella; S, starch; M, mitochondria; N, nucleus; NL, nucleolus; P, plastid; S, starch; M, mitochondria; V, vacuole; ER, Endoplasmic reticulum; GA, Golgi bodies.

To further investigate the effect of ALA on organelles such as chloroplast, ultrastructure and morphometric images were used. Changes in the whole mesophyll cells and chloroplasts are shown in Figure 4. Seedlings grown under normal conditions exhibited regular cell shape and typical chloroplast. There are smoothly arrayed grana lamellae and a small quantity of osmiophilic granules (Figures 4A,a–d). The cell morphological disturbance and plasmolysis occurred when seedlings were treated with 50 μM CdCl_2_, but the number of mitochondria markedly increased (Figures 4A,i–l). The grana lamellae of thylakoid were loose, and the shapes of chloroplasts were severely swollen. Furthermore, there were plenty of osmiophilic granules in the chloroplast (Figures 4A,i–l). For the ALA-treated seedlings under Cd stress condition, although there was a little improvement in cell morphology, the shapes of chloroplast become typically fusiform (Figures 4A,m–p). Moreover, the chloroplasts contained more orderly grana lamellae and starch grains and fewer osmiophilic granules (Figures 4A,p,l). Under normal growth conditions, ALA-treated seedlings were very similar to those of the control (Figures 4A,e–h).

The ultrastructure of root tip cells with low and high magnifications is shown in Figure 4B. Seedlings grown in normal conditions are shown in Figures 4B,a–d, and the root cells had regular cell morphology. The cells contained endoplasmic reticulum and well-shaped mitochondria with large vacuoles in the center. The mitochondria had a clear inner membrane structure and were concentrated near the plastids. Under 50 μM Cd treatment, the cells exhibited obvious ultrastructural changes (Figures 4B,i–l). The cell wall was thickened, and plasmolysis was observed. The number of plastids and starch grains decreased; however, the number of mitochondria increased. The Cd existed in the form of small granules along the cell wall. The micrographs of root tip cells that were simultaneously treated with 25 mg/L ALA and 50 μM Cd were analyzed (Figures 4B,m–p). The large central vacuole was visible. The ion deposition of cytoplasm decreased, and the plastids and starch grains were few. However, the number of mitochondria increased. The ALA improved the cell structure of root tips, which showed various developed mitochondria. These results confirmed that ALA can ameliorate the effect of Cd stress. The micrographs of root tip cells under ALA treatment alone (Figures 4B,e–h) showed regular cell morphology and normal organelle structure, which were similar to that of the control group.

### 5-Aminolevulinic Acid Alleviated Oxidative Damage Under Cadmium Stress

**FIGURE 5:**
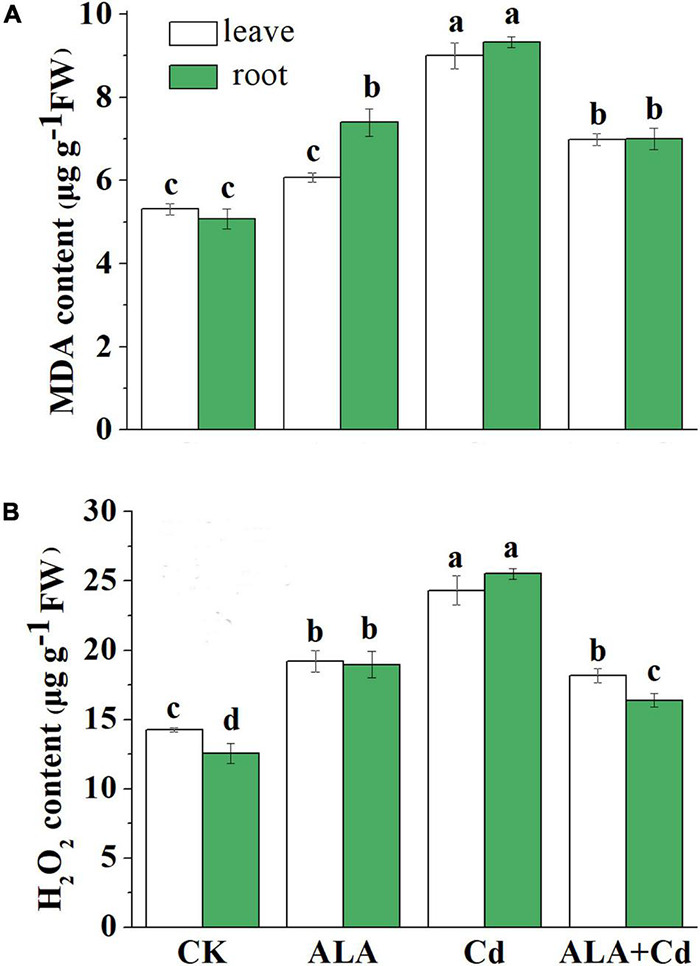
Effects of ALA under Cd stress on contents of MDA **(A)** and H_2_O_2_ **(B)** of cabbage. The seedlings were treated as in Figure 1. The mean ± SE are shown (*n* ≥ 3). Different letters indicate significant difference among the treatments (*P* < 0.05).

The Cd stress inevitably induces H_2_O_2_ and MDA productions in plants (Figure 5A). Results showed that Cd stress significantly increased MDA content in the leaves and roots, and it reached 170 and 184% of the control, respectively. The application of exogenous ALA can reduce the MDA content of leaves and roots under Cd stress. While spraying ALA under normal conditions, the content of MDA in the leaves and roots is similar to the control level.

As shown in Figure 5B, the Cd stress significantly increased H_2_O_2_ content in cabbage leaves and roots, which reached 171 and 203% of the control, respectively. Exogenous application of ALA can significantly downregulate H_2_O_2_ content in the roots of stressed plants. It was decreased by 25 and 36% in the leaves and roots, respectively, compared to that under Cd treatment (Figure 5B). All these results showed that ALA can reduce the toxicity of Cd by reducing oxidative damage.

### Effects of Exogenous 5-Aminolevulinic Acid on the Enzymes’ Activities of Ascorbate-Glutathione Cycle in Cabbage

**FIGURE 6:**
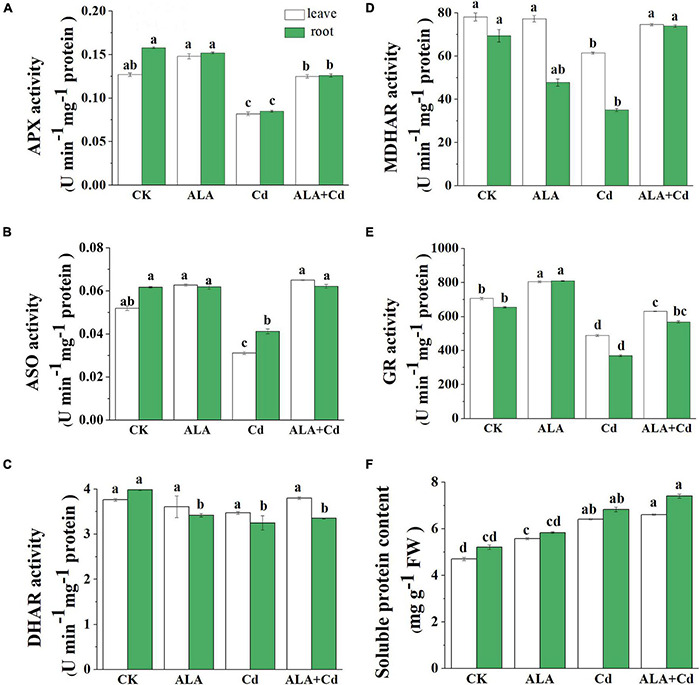
Effects of ALA under Cd stress on the key enzyme activities of AsA-GSH cycle in cabbage. **(A)** APX; **(B)** ASO; **(C)** DHAR; **(D)** MDHAR; **(E)** GR; **(F)** Soluble protein. The seedlings were treated as in Figure 1. The mean ± SE are shown (*n* ≥ 3). Different letters indicate significant difference among the treatments (*P* < 0.05).

Under Cd stress, MDA and H_2_O_2_ content were dramatically increased, which caused oxidative stress in Chinese cabbage. The enzyme activities involved in the AsA-GSH cycle, which is the main way of scavenging H_2_O_2_, were analyzed in cabbage roots and leaves. Results showed that the activity of APX decreased slightly by 35 and 46% under Cd stress in leaves and roots, respectively (Figure 6A). The activity of ASO was also inhibited in leaves and roots and decreased by 40 and 33%, respectively (Figure 6B). Under the ALA + Cd treatment, the APX and ASO activities increased significantly by 52 and 108% in leaves and by 48% and 51% in roots, respectively.

Moreover, the Cd stress had no significant difference in the DHAR activity both in leaves and roots (Figure 6C). The activity of MDHAR was increased by 21 and 111% in leaves and roots, respectively, under ALA + Cd stress (Figure 6D). The GR activity can be inhibited by Cd stress (Figure 6E), and exogenous ALA treatment significantly increases GR activity, and it reached 129 and 153% in leaves and roots, respectively, of Cd treatment alone. The soluble protein content was significantly increased under Cd treatment alone as well as ALA + Cd treatment; however, there is no significant difference between Cd treatment alone and ALA + Cd treatment (Figure 6F).

### Effects of 5-Aminolevulinic Acid on Ascorbic Acid and Glutathione Contents in Cabbage

**FIGURE 7:**
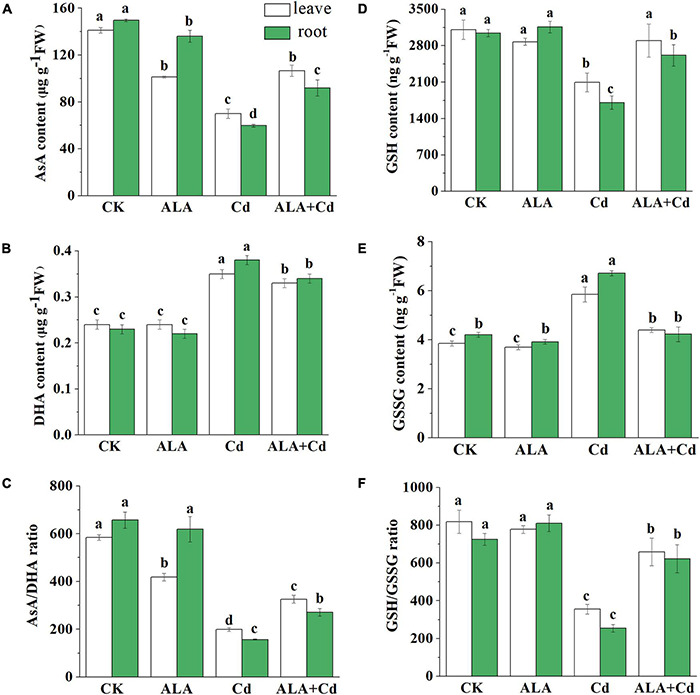
Effects of ALA under cadmium stress on contents of AsA, DHA, GSH, and GSSG in cabbage. **(A)** AsA; **(B)** DHA; **(C)** AsA/DHA; **(D)** GSH; **(E)** GSSG; **(F)** GSH/GSSG. The seedlings were treated as in Figure 1. The mean ± SE are shown (*n* ≥ 3). Different letters indicate significant difference among the treatments (*P* < 0.05).

As antioxidants, AsA and glutathione (GSH) play an important role in scavenging free radicals through the AsA-GSH cycle. To further verify the protective mechanism of ALA in cabbage tolerance to Cd stress, the AsA and GSH content were measured (Figure 7). The AsA content was significantly decreased by 51 and 60% in the leaves and roots of Chinese cabbage under Cd stress, respectively (Figure 7). The content of AsA was increased by approximately 53 and 54% in leaves and roots under the application of exogenous ALA under Cd stress, respectively. Contrary to the changes of AsA content, the DHA significantly increased about 46 and 65% in leaves and roots under Cd stress. The contents of DHA in ALA and Cd co-treatment were similar to that of Cd treatment. In addition, there was no difference in the DHA contents of the plant between control conditions and ALA treatment (Figure 7B).

Results showed that GSH content was significantly reduced by 33 and 44% in leaf and root, respectively, under Cd stress (Figure 7D). The change of GSSG content was opposite to that of GSH. The Cd stress markedly increased the GSSG content. However, the application of exogenous ALA reduced the GSSG content by 24.8 and 37.1% in leaves and roots under ALA + Cd co-treatment, respectively (Figure 7E).

Figure 7C showed that the ratio of AsA/DHA was significantly lower than that of CK condition under Cd stress. While the exogenous application of ALA can significantly improve AsA/DHA value. In addition, similar to the above results, the GSH/GSSG ratio was also decreased under Cd stress (Figure 7F), and spraying ALA under Cd stress could increase the ratio of GSH/GSSG.

### Effects of 5-Aminolevulinic Acid on Accumulations of Mineral Elements in Cabbage

**FIGURE 8:**
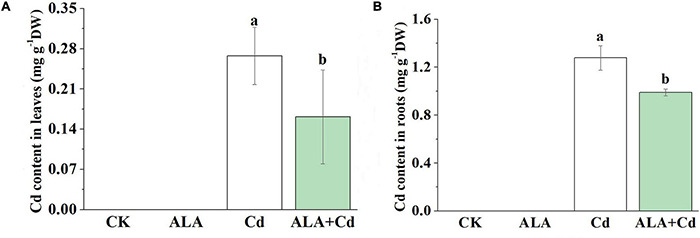
Effects of ALA under Cd stress on content of Cd in leaves **(A)** and roots **(B)**. The seedlings were treated as in Figure 1. The mean ± SE are shown (*n* ≥ 3). Different letters indicate significant difference among the treatments (*P* < 0.05).

The Cd toxicity alters the absorption of mineral nutrition by plants (Khan et al., 2015). Foliar application of ALA not only significantly decreased Cd accumulation in leaves (by 40%) and roots (by 23%) of Chinese cabbage (Figures 8A,B) but also affected the uptake of other elements by plants. Compared to the control, Cd treatment significantly reduced the levels of the nutrient in plants (Tables 2, 3). It has also reduced the contents of Ca, Mg, Fe, Zn, and Mn by 49, 60, 47, 39, and 53% in cabbage leaves, respectively. Additionally, Cd treatment reduced the content of Fe and Mn by 33 and 59% in roots, respectively, but there was no difference in levels of other elements in the roots. The contents of the mineral elements in the ALA and Cd co-treated plants were lower than those of control plants, but they were generally higher than that of plants treated with Cd alone. After the exogenous application of ALA, the contents of nutrient elements in leaves and roots were similar to those of control plants.

**TABLE 2:** Effects of ALA on the concentrations of Fe^2+^, Zn^2+^, Mn^2+^, Ca^2+^, and Mg^2+^ in leaves under Cd stress.

Treatment mg g^–1^ DW	Fe	Zn	Mn	Ca	Mg
CK	0.1989 ± 0.0058a	0.3625 ± 0.0057a	0.3312 ± 0.0301a	2.4845 ± 0.0017a	3.8175 ± 0.0045a
ALA	0.1828 ± 0.0150a	0.3434 ± 0.0092a	0.2620 ± 0.0425a	2.5440 ± 0.0012a	3.6790 ± 0.0081a
Cd	0.1055 ± 0.0115b	0.2212 ± 0.0100b	0.1550 ± 0.0405b	1.2765 ± 0.0002c	1.5455 ± 0.0089c
Cd+ALA	0.1820 ± 0.0131a	0.3304 ± 0.0034a	0.2152 ± 0.0333a	2.2790 ± 0.0024ab	3.4285 ± 0.0029ab

*Different letters indicate significant difference among the treatments.*

**TABLE 3:** Effects of ALA on the concentrations of Fe^2+^, Zn^2+^, Mn^2+^, Ca^2+^, and Mg^2+^ in roots under Cd stress.

Treatment mg g^–1^ DW	Fe	Zn	Mn	Ca	Mg
CK	0.7472 ± 0.0370a	0.6682 ± 0.0054a	0.3767 ± 0.0059a	0.6265 ± 0.0030a	1.1992 ± 0.0091a
ALA	0.7718 ± 0.0294a	0.5859 ± 0.0103a	0.3704 ± 0.0020a	0.6300 ± 0.0015a	1.2461 ± 0.0012a
Cd	0.5007 ± 0.0122b	0.6273 ± 0.0084a	0.1538 ± 0.0049c	0.6065 ± 0.0010a	1.0728 ± 0.0074a
Cd+ALA	0.6531 ± 0.0128a	0.6961 ± 0.0057a	0.2515 ± 0.0127ab	0.6395 ± 0.0035a	1.0497 ± 0.0484a

*Different letters indicate significant difference among the treatments.*

### Effects of 5-Aminolevulinic Acid on the Expression of Genes Involved in Cadmium Transport

**FIGURE 9:**
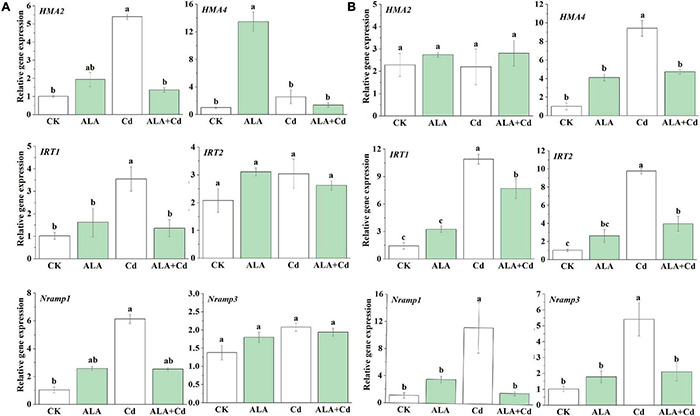
The qRT-PCR analysis of the expression of genes involved in ion absorption and transport in Chinese cabbage seedlings in leaves **(A)** and roots **(B)**. The relative expression levels of genes are normalized to *Actin*. The seedlings were treated as in Figure 1. The mean ± SE are shown (*n* ≥ 3). Different letters indicate significant difference among the treatments (*P* < 0.05).

Cadmium is absorbed from the soil by the roots, loaded into the xylem, and transported to the ground by several transporters (Han et al., 2014). Iron-regulated transporter 1 (*IRT1*) and *IRT2* are the major transporters for Fe uptake (Vert et al., 2002), and they can also transport several other divalent metals, i.e., Fe, Zn, Mn, and Cd (Rogers et al., 2000). Both *Nramp1* and *Nramp3* participated in Cd transportation *via* the vacuole tonoplast (Lin and Aarts, 2012). The *HMA2* and *HMA4* are responsible for Cd translocation and distribution (Mill et al., 2003; Elif and Argüello, 2004). The effects of ALA on the ion uptake and the translocation-related genes under Cd stress were investigated. Using qRT-PCR technology, the expression level of *HMA2* and *HMA4* genes in the shoots of Chinese cabbage seedlings under Cd stress was significantly increased by 5.2 and 13.3 times, respectively, in the roots; only *HMA4* expression increased and *HMA2* expression showed no changes (Figure 9). The transcription level of *HMA4* genes in shoots and roots were decreased significantly under ALA + Cd treatment compared with that under Cd treatment, respectively, while *HMA2* decreased by 75% in shoots.

The expression level of the *IRT1* and *IRT2* genes in the roots of seedlings under Cd stress was significantly increased by 7.6 and 9.5 times, respectively, while in the shoots, only the *IRT1* expression increased, and *IRT2* expression showed no change. The transcription levels of *IRT1* and *IRT2* genes were significantly decreased by 1.4 and 2.5 times under ALA and Cd treatment, respectively, in the roots, while *IRT1* expression was decreased by 2.6 times in shoots. Similarly, the Cd stress can upregulate the transcription level of *Nramp1* and *Nramp3* in the roots of Chinese cabbage, and *Nramp1* was also regulated in shoots. The transcription level of these genes was significantly increased under Cd stress (Figures 9A,B). The exogenous application of ALA can significantly decrease the expression of genes under Cd stress. Overall, there was no significant effect on these genes when the exogenous ALA was applied under the control condition. Most genes were significantly upregulated under Cd stress in shoots or roots. There were significantly downregulated in the ALA + Cd treatment compared with Cd treatment alone (Figure 9). These results indicated that ALA could affect Cd uptake and accumulation of Chinese cabbages by downregulating the transcription of Cd uptake and transfer-related genes.

## Discussion

The Cd can accumulate in the edible part of crops, and ultimately enter the food chain (Hu et al., 2013; Song et al., 2016; Rizwan et al., 2017). The Cd toxicity causes cell death, leaf roll, and chlorosis, and diminishes plant growth biomass and yield (Mombo et al., 2016). The Cd stress reduced the biomass of cabbage and shortened the primary root length (Figure 1). In the former studies, ALA has been proven as a new plant growth regulator due to its effects on plants at the physiological, biochemical, and molecular levels (An et al., 2019). The ALA can be effective against the harmful effects caused by abiotic stress in plants (Aksakal et al., 2017). In this study, we aimed to investigate the protective mechanism of ALA involved in Cd stress in Chinese cabbage. In this study, Cd stress was found to decrease the Chl content in Chinese cabbage (Figure 2). However, ALA effectively alleviated Cd stress and increased the biomass, the length of primary roots, and the Chl contents of cabbage. The exogenous application of ALA increased the biomass and leaf Chl contents of *Brassica napus* L. seedlings under Cd stress (Xu et al., 2016). These results confirmed the protective effects of ALA in the tolerance of cabbage plants to Cd stress.

The Cd stress caused oxidative stress in vegetables by inducing the excessive accumulation of H_2_O_2_ (Gratão et al., 2015). The MDA is the main product of membrane system peroxidation, and it is an important index of membrane lipid peroxidation. In this study, Cd increased H_2_O_2_ and MDA content in cabbage leaves and roots. Exogenous application of ALA significantly decreased H_2_O_2_ and MDA contents to the control level (Figure 5). It has been reported that ALA can reduce MDA and H_2_O_2_ levels by enhancing the gene expressions of antioxidant enzymes in *Brassica napus* under Cd stress (Ali et al., 2013a,^2015^; Wu et al., 2015, 2017). These findings provided evidence that ALA can improve the tolerance of cabbage to Cd by decreasing oxidative damage. Plants have enzymatic and non-enzymatic systems that respond to ROS in the cell when exposed to stresses, thus, reducing the damage to plant cells (Wang and Tam, 2018). The enzymatic and non-enzymatic systems can be triggered in response to Cd-induced oxidative stress, which involves a set of antioxidant enzymes and non-enzymes antioxidants. The AsA-GSH is an important active oxygen scavenging system in plants (Kaya et al., 2020). This study revealed that Cd toxicity decreased the activities of enzymatic antioxidants (APX, ASO, DHAR, MDHAR, and GR) in cabbage seedlings (Figure 6). Meanwhile, the application of ALA stimulated the activities of major enzymes in the AsA-GSH system. The AsA and GSH regulate plant metabolism by promoting the removal of ROS and relieving the oxidative damage on plants caused by stresses (Gill and Tuteja, 2010). It was reported that foliar application of ALA in sunflower hybrid can reduce drought-induced yield losses and improve oil content by improving the activities of catalase, superoxide dismutase, and ascorbate peroxidase (Sher et al., 2021). Under Cd stress, DHA and GSSG levels increased, but AsA and GSH levels decreased. The exogenous application of ALA promoted the exchange of DHA to AsA and increased the content of GSH and the GSH/GSSG ratio (Figure 7). Thus, the antioxidant capacity and redox balance of plants under stresses regulated by ALA is crucial. These results, along with previous findings (Wu et al., 2019) showed that ALA application could improve the tolerance of Chinese cabbage to Cd by increasing the non-enzymatic antioxidant level and the activity of antioxidant enzymes.

The structural integrity of the chloroplast is essential to maintaining normal growth and photosynthesis, and it can be easily subjected to oxidative stress under stress conditions. A Pn indicates the strength of organic matter accumulated through photosynthesis in plants (Lucas et al., 2013). Electron microscopy technology was used to analyze ultrastructural changes and investigate the mechanism of biosynthesis under Cd stress. Cell structure was damaged when treated with 50 μM Cd, for example, the cell wall, cell membrane intercellular spaces, and increased size and number of starch grains were observed in ruptured chloroplasts. The thylakoid membrane was dispersed and elongated under Cd treatment alone, and there was an increased number of lipid bodies (Figure 4A). Meanwhile, Cd stress caused Pn, Gs, and Tr to decrease and Ci to increase (Figure 2). The increase in starch grains under Cd stress might be due to nutrient deficiency (Daud et al., 2009). Chloroplast was pronounced and was visible upon ALA application, and there was less starch grain and plastoglobuli (Figure 4A). Exogenous ALA can alleviate the toxic effects of Cd in the mesophyll cell and improve the cell structure (Ali et al., 2015). Exogenous ALA can reduce the lipid bodies in the cucumber cytoplasm under salt stresses (Wu et al., 2018). The reduction in the number of lipid bodies and plastoglobuli in chloroplasts is an indication of lesser oxidative stress. Zhang et al. (2008) concluded that ALA reduced the lipid peroxidation of thylakoids by inducing the antioxidant system. The application of biological fertilizer [Arbuscular Mycorrhizal Fungi (AMF)] also decreased the cell and grain size and improved the tolerance of soybean to drought stress (Sheteiwy et al., 2021). In this study, we found that ALA improved cell form and chloroplast structure when treated either with ALA alone or in combination with Cd conditions. The ALA also significantly increased Pn, Gs, and Tr and reduced the Ci value in cabbage.

The content of Chl is directly related to the intensity of photosynthesis (Cao et al., 2013). The Cd significantly reduced Chl contents and Fv/Fm in Chinese cabbage plants (Figure 3). The decreased levels of Chl content and Fv/Fm might inhibit photosynthesis by affecting the regeneration and degradation of photosynthetic organs and reducing the photo energy conversion efficiency. These results are similar to the effects of low temperatures (Chen et al., 2017) or high temperatures (Wang D. Y. et al., 2018). The ALA treatment also enhanced Fv/Fm and qP under Cd stress, meanwhile, both Chl a and Chl b content were increased. The ALA can enhance Chl biosynthesis under stresses by upregulating gene expression of glutamyl-tRNA reductase (*HEMA1*), Mg-chelatase (*CHLH*), and protochlorophyllide oxidoreductase (*POR*) in the Chl synthesis pathway (Wu et al., 2018). The ALA treatment significantly promoted the electron transfer activity of the PSII reaction center on the donor side, the reaction center itself, and the receptor site and increase the intensity of photosynthesis (Yang et al., 2021). From the above results, we concluded that exogenous ALA could repair the mesophyll cell structure, increase the utilization of CO_2_, improve photosynthesis, and ultimately increase the biomass of cabbage.

The Cd stress may be attributed to the competition between nutrients and Cd for the same transporters, which inevitably disturbed the balance of mineral elements (Ramos et al., 2002). The Cd was found to impair the nutrient balance and accumulation (Matraszek et al., 2016; Wu et al., 2017). In oilseed rape and lettuce, Cd stress caused an imbalance of mineral elements (Xu et al., 2016; Dawuda et al., 2020). It was found that AMF could increase the nutrient absorption of Guar (El-Sawah et al., 2021). In this experiment, the Fe content significantly declined in leaves and roots of cabbage under Cd stress (Tables 2, 3). The ALA application increased Fe content in leaves and roots under Cd stress to the control level. The results showed that ALA had little effect on the contents of Zn, Ca, and Mg in the roots. Moreover, ALA improved the concentration of nutrients under Cd stress in *Brassica napus* (Xu et al., 2016). It has been found that ALA alleviated the decrease of N, P, Ca, Mg, Zn, and Fe content in rape (*Brassica napus*) under salt stress, but had no effect on Mn and Cu contents (Naeem et al., 2010). However, Liu et al. (2017) did not observe an increase in K, Fe, and Mg content alongside the improvement in alkaline tolerance of Swiss chard (*Beta vulgaris* L.) treated with ALA. Therefore, ALA can inhibit Cd uptake and improve Fe and Mn translocation from roots to leaves in cabbage. Thus, the effect of ALA on the content of mineral elements in plants may be related to species and types of stress.

The Cd-toxicity alternates the absorption of mineral nutrition for plants (Alexandre et al., 2012). The ALA could significantly reduce the Cd content in the roots and leaves of cabbage. Both *IRT1* and *IRT2* are expressed in the external cell layers of roots, specifically under Fe starvation (Vert et al., 2002). Wang reported that exogenous melatonin (MT) can decrease the expression of *IRT1* and Cd content (Wang et al., 2020). In this study, we found that ALA affected the absorption of Cd by decreasing the expression of *IRT1* and *IRT2*, thereby decreasing the content of Cd in the roots of Chinese cabbage (Figure 8). Similar results have been obtained in other studies. The exogenous hydrogen-rich water reduced Cd levels by suppressing the expression of *IRT1* in *Arabidopsis thaliana* (Wu et al., 2021). The *AtNramp1* can cooperate with *AtIRT1* to take up Fe^2+^ in Arabidopsis roots (Chen et al., 2019). It can be concluded that *Nramp* genes were indispensable for balanced element absorption in plants. It has been shown that overexpression of *HMA* plays a role in Cd accumulation (Takahashi et al., 2012). In this study, we showed that ALA could significantly inhibit the upregulation of *IRT1, IRT2*, *Nramp1*, *Nramp3, HMA2*, and *HMA4* transcription induced by Cd stress, which was consistent with the effect of ALA on Cd levels (Figure 9). Therefore, it can be inferred that spraying ALA on leaves may affect Cd uptake by downregulating the transcription of *IRT1* and *IRT2*, and inhibiting the activation of Cd in vacuole by affecting the transcription of *Nramp1* and *Nramp3*, thereby reducing the effective cytotoxic Cd concentration. The decrease in *HMA2* and *HMA4* transcription inhibits the transfer of Cd from root to shoot and improves the tolerance of Chinese cabbage to Cd stress.

## Conclusion

**FIGURE 10:**
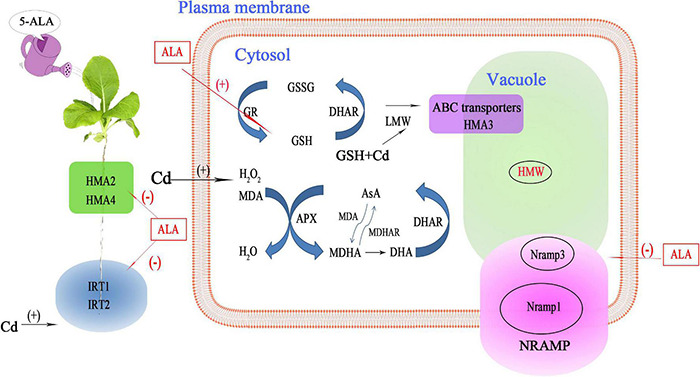
A model illustrating the role of ALA in Chinese cabbage seedlings’ tolerance to Cd stress. Cd stress increased the content of MDA and H_2_O_2_ in Chinese cabbage seedlings. The ALA can increase the enzyme activities of the antioxidant system, induce a large amount of GSH and AsA, and remove ROS and Cd ions in plants. Meanwhile, ALA downregulates the expression of the Cd absorption and transportation-related genes (*HMA2, HMA4, Nramp1*, and *Nramp3*), which ultimately leads to a decrease in Cd level and an increase in resistance to Cd stress. This process increases the tolerance of Chinese cabbage to Cd. (+) Positive regulation; (–) Negative regulation.

In summary, exogenous ALA treatment can effectively reduce the Cd accumulation in Chinese cabbage by eliminating excess ROS and regulating Cd-induced transport genes. These results highlighted the potential function of ALA in combating Cd stress (Figure 10). According to the results, Cd treatment seriously inhibited the photosynthesis in cabbage, decreased antioxidant capacity, disturbed the nutrients balance, and resulted in oxidative stress. Exogenous ALA can promote the response of cabbage to Cd stress by enhancing the efficiency of light-to-energy conversion, improving Chl content, and maintaining high redox homeostasis by scavenging ROS and regulating the antioxidant enzyme activities in the AsA-GSH cycle. Moreover, ALA can also downregulate the expression of transport genes that mediate the binding to Cd, and, thus, reducing the Cd content. These changes in the physiological state and gene transcriptions reflect the adaptation of plant response to Cd stress under the exogenous ALA application. To study the precise mechanism of reduced Cd accumulation in cabbage due to ALA treatment, an environment-based design of soil research is required.

## Data Availability Statement

The original contributions presented in the study are included in the article/supplementary material, further inquiries can be directed to the corresponding author.

## Author Contributions

LY, JL, and JY conceived and designed the research. LY and RW conducted the experiments. LY, XW, and ZT analyzed the data and prepared the figures and illustrations. LY wrote the manuscript. YW, SL, and LH read the manuscript and made valuable inputs. XW and BA read the manuscript and checked the language grammar. All authors read and approved the submission of the manuscript.

## Conflict of Interest

The authors declare that the research was conducted in the absence of any commercial or financial relationships that could be construed as a potential conflict of interest.

## Publisher’s Note

All claims expressed in this article are solely those of the authors and do not necessarily represent those of their affiliated organizations, or those of the publisher, the editors and the reviewers. Any product that may be evaluated in this article, or claim that may be made by its manufacturer, is not guaranteed or endorsed by the publisher.
